# Domain Adaptation for Bearing Fault Diagnosis Based on SimAM and Adaptive Weighting Strategy

**DOI:** 10.3390/s24134251

**Published:** 2024-06-30

**Authors:** Ziyi Tang, Xinhao Hou, Xinheng Huang, Xin Wang, Jifeng Zou

**Affiliations:** 1 School of Electrical Engineering and Automation, Tianjin University of Technology, Tianjin 300384, China; ziyitang2003@gmail.com (Z.T.); houxinhao@stud.tjut.edu.cn (X.H.); 2086708672@stud.tjut.edu.cn (J.Z.); 2 Maritime College, Tianjin University of Technology, Tianjin 300384, China; hxh@stud.tjut.edu.cn; 3 Engineering Training Center, Tianjin University of Technology, Tianjin 300384, China; 4 Institute of Intelligent Control and Fault Diagnosis, Tianjin University of Technology, Tianjin 300384, China

**Keywords:** bearing fault diagnosis, domain adaptation, adaptive weighting strategy, CWT, SimAM

## Abstract

Domain adaptation techniques are crucial for addressing the discrepancies between training and testing data distributions caused by varying operational conditions in practical bearing fault diagnosis. However, transfer fault diagnosis faces significant challenges under complex conditions with dispersed data and distinct distribution differences. Hence, this paper proposes CWT-SimAM-DAMS, a domain adaptation method for bearing fault diagnosis based on SimAM and an adaptive weighting strategy. The proposed scheme first uses Continuous Wavelet Transform (CWT) and Unsharp Masking (USM) for data preprocessing, and then feature extraction is performed using the Residual Network (ResNet) integrated with the SimAM module. This is combined with the proposed adaptive weighting strategy based on Joint Maximum Mean Discrepancy (JMMD) and Conditional Adversarial Domain Adaption Network (CDAN) domain adaptation algorithms, which minimizes the distribution differences between the source and target domains more effectively, thus enhancing domain adaptability. The proposed method is validated on two datasets, and experimental results show that it improves the accuracy of bearing fault diagnosis.

## 1. Introduction

With the advent of Industry 4.0, modern information technology has become deeply integrated with manufacturing, leading to significant advancements in machine manufacturing and industrial production. Rotating machinery equipment is extensively used in these fields, and bearings, as key mechanical components of such machinery, affect the safe operation of the equipment in its entirety [1,2,3]. Statistics show that about 40% of failures in rotating equipment are caused by bearing faults [4]. Thus, accurate and real-time detection of bearing faults is essential for the smooth progress of mechanical manufacturing and industrial production.

With the boom in big data and artificial intelligence technology, data-driven intelligent fault diagnosis methods have become a key research focus in recent years [5,6,7]. Data processing plays a key role in the effectiveness of fault diagnosis. Raw bearing fault signals typically reflect time domain information, and, after serial processing, they can reveal frequency domain information. However, considering only time or frequency domain information, the model’s fault diagnosis performance is often suboptimal when dealing with nonlinear bearing fault diagnosis signals. Therefore, attention has been given to the Continuous Wavelet Transform (CWT), which can simultaneously reflect both time and frequency domain information. Gu et al. [8] proposed a hybrid deep learning model for fault diagnosis that effectively extracts fault features from bearings and handles small sample datasets. This model uses variational modal decomposition (VMD) [9] and CWT algorithms for data processing and employs a convolutional neural network (CNN) [10] for model training. Cheng et al. [11] introduced a rotational machinery diagnosis method based on the CWT and Local Binary Convolutional Neural Networks. Stable and accurate fault diagnosis technology can reliably detect the types of faults in motors, providing reliable support for the operational monitoring and maintenance of rotating machinery [5,6,7]. As an intelligent algorithm, the deep learning model can extract fault features from bearing data for end-to-end fault diagnosis. Wang et al. [12] proposed a method combining an improved residual network and wavelet transform for intelligent gearboxes. This approach effectively extracts features and diagnoses single faults, compound faults, and unbalance faults. Jiang et al. [13] introduced a multi-scale convolutional neural network featuring channel attention utilizing both max pooling and average pooling layers to identify bearing fault characteristics at different scales. Regarding nonlinear feature extraction methods, Zhang et al. [14] developed an adaptive activation function with a *tanh* function and slope thresholding. These were incorporated into the Residual Network (ResNet), allowing the network to extract features that are significantly different between faults types. However, deep learning requires a large number of data for training, and deep learning algorithms need training and testing data to have the same operational conditions, meaning they must share the same distribution. Real-time changes in operating conditions such as humidity, voltage, speed, current fluctuations, and load can cause data distribution variations during the normal operation of actual rotating equipment. These changes decrease the accuracy of deep learning algorithms when processing test dataset data [15].

Recently, bearing fault diagnosis methods based on domain adaptation transfer learning have addressed several challenges. Specifically, they have resolved the issues of low generalizability and low robustness owing to limited data in deep learning. They have tackled the problems associated with the source and target data being in different feature spaces or distributions. Schwendemann et al. [16] proposed the Layered Maximum Mean Discrepancy (LMMD) method, an extension of the Maximum Mean Discrepancy (MMD) that incorporates the unique characteristics of the proposed intermediary domain. Lu et al. [17] developed an architecture in which the conditional and marginal distributions are adapted across multiple neural network layers. This method uses the MMD to measure the distribution discrepancies and introduces an adaptive weighting strategy to ascertain the importance of different distributions. Mao et al. [18] combined the adaptability of Domain Adversarial Neural Networks (DANNs) with structured relational information across various failure models to enhance transfer learning effectiveness. Chen et al. [19] proposed the Multi-Gradient Hierarchical Domain Adaptation Network, which concurrently acquires transferable domain invariance and class-discriminative insights, improving the diagnostic transferability of bearing faults. All of these methods have achieved satisfactory results in some respects. However, traditional bearing fault diagnosis methods based on transfer learning and CWT time–frequency images still face the following major challenges in feature capture and domain adaptation:

(1) When the fault signal is weak, the data are smooth, or feature contrast is not apparent. CWT alone may not clearly display bearing fault characteristics. Therefore, enhancing data contrast through sharpening methods to improve the discriminative power of data features is particularly important.

(2) In the process of feature extraction for fault diagnosis, models need strong feature capture capabilities. Traditional residual networks often struggle to adequately focus on important features when capturing complex fault patterns, leading to suboptimal feature extraction.

(3) Domain adaptation algorithms based on kernel methods, such as the MMD, LMMD, and Joint Maximum Mean Discrepancy (JMMD), rely heavily on the selection and tuning of the kernel function to achieve feature alignment. When dealing with data exhibiting complex nonlinear distributions, the choice of kernel function greatly influences the algorithm’s ability to capture feature differences and interactions within the data. Domain adaptation algorithms based on adversarial learning, such as DANNs and Conditional Adversarial Domain Adaption Networks (CDANs), align features between the source and target domains through adversarial training. Although adversarial learning excels at capturing complex nonlinear distribution differences, the training process is prone to gradient instability, mode collapse, and vanishing gradients, making it difficult for the model to converge. Additionally, a DANN primarily focuses on aligning feature distributions and lacks explicit alignment of class conditions, which can adversely affect classification performance.

In order to solve the problems mentioned above, this paper proposes the CWT-SimAM-DAMS model. The specific innovations and contributions are as follows:

(1) The one-dimensional bearing fault signal is intergrated using a sliding window, the segmented data are processed with the CWT algorithm, and, finally, the resulting CWT time–frequency images are enhanced by overlaying high-frequency features using the Unsharp Masking (USM) algorithm. This method is named CWT-USM.

(2) The SimAM attention mechanism is integrated into the Residual Network to enhance the model’s feature extraction capability for input images and provide a robust feature extraction foundation for JMMD and CDAN domain adaptation algorithms. This model is named SimAM-ResNet.

(3) The model’s generalization ability is enhanced utilizing the JMMD and CDAN domain adaptation algorithms and designing an adaptive weighting strategy. The JMMD domain adaptive algorithm provides stable distribution alignment to make adversarial training more stable, and the CDAN domain adaptive algorithm mitigates the JMMD domain adaptive algorithm’s dependence on the kernel method by capturing complex nonlinear distribution differences through adversarial learning. CDAN and JMMD domain adaptive algorithms focus on both the joint distribution of labels and features. The adaptive weighting strategy considers the classification, JMMD, and CDAN loss, effectively reducing the discrepancy in joint distributions and achieving global domain alignment. Additionally, parameters are adaptively adjusted at various stages of model training to ensure the model’s optimal performance.

The rest of the paper is organized as follows: Section 2 describes the theoretical concepts of transfer learning, CWT, USM, SimAM, and ResNet. Section 3 presents a new domain adaptive method for diagnosing bearing faults, including the SimAM attention mechanism, the JMMD and CADN domain adaptation algorithms, and the weight adaptive strategy. Section 4 describes the specifics of the dataset and the parameter settings used in this study. Section 5 provides experimental results and conducts an analysis. Section 6 summarizes the paper.

## 2. Theoretical Background

### 2.1. Description of Transfer Learning Problems

In domain adaptation [5], the source domain is defined as $D_{s}=\left\{\chi^{s},P\left(x_{s}\right)\right\}$ and the target domain as $D_{t}=\left\{\chi^{t},P\left(x_{t}\right)\right\}$. The dataset for the source domain is $X^{s}={\left\{\left(x_{i}^{s}\right)\right\}}_{i=1}^{N_{s}}$, with labels $Y^{s}={\left\{\left(y_{i}^{s}\right)\right\}}_{i=1}^{N_{s}}$, where $y_{i}\in 1,2...,K$, and *K* denotes the total number of categories. The dataset for the target domain is $X^{t}={\left\{\left(x_{i}^{t}\right)\right\}}_{i=1}^{N_{t}}$. The main problem addressed in this paper is that the feature space of the source and target domains are the same, i.e., $\chi^{s}=\chi^{t}$, but their marginal distributions differ, i.e., $P\left(x_{s}\right)\neq P\left(x_{t}\right)$.

### 2.2. Continuous Wavelet Transform

When dealing with the continuous one-dimensional vibration signals of motor faults, an effective feature extraction strategy is to convert the signals into two-dimensional time–frequency images. This method not only enriches the representation of frequency domain information, but also makes it more suitable for the learning process of neural networks due to its two-dimensional structure. In the time–frequency plots, one can directly observe the changes in signal frequency components over time. The CWT is particularly adept due to its window scaling, which overcomes the limitations of the Short-Time Fourier Transform (STFT) [20,21], where window sizes do not vary with frequency or time, making it more suitable for handling transient signals like those in motor faults. The CWT is mathematically formulated as follows [22]:

(1) $$W_{x}\left(a,b\right)=\frac{1}{\sqrt{a}}\int_{-\infty }^{+\infty }x\left(t\right)\psi^{*}\left(\frac{t-b}{a}\right)dt$$

where $W_{x}\left(a,b\right)$ represents the wavelet coefficients, *a* is the scaling parameter, and *b* is the translation parameter. The choice of the mother wavelet is crucial in wavelet transforms as it determines the accuracy and efficiency of the transform. Common mother wavelets include the Daubechie Wavelet, Reverse Biorthogonal Wavelet, Bior Wavelet, and Morlet Wavelet. This paper selects the cmor wavelet as the mother wavelet for the CWT because the cmor wavelet, a complexified version of the Morlet Wavelet, possesses excellent time–frequency localization properties and effective filtering and signal reconstruction capabilities.

### 2.3. Unsharp Masking

Unsharp Masking is a widely used technique for sharpening enhancement. The USM algorithm acquires high-frequency components by subtracting the low-pass filtered blurred image from the original image. These high-frequency parts are then multiplied by a gain coefficient and added back to the original image, enhancing the contrast of these high-frequency components and thereby improving the visual clarity of the image details and edges in the image. The processing steps of Unsharp Masking are as [23] follows:

**Step 1:** Use a Gaussian filter to create a blurred version of the original image and reduce its high-frequency content.

(2) $$G\left(x,y,\sigma \right)=\frac{1}{2\pi \sigma^{2}}e^{-\frac{x^{2}+y^{2}}{2\sigma^{2}}}$$

where *x* and *y* denote the positions relative to the center pixel, and σ is the standard deviation of the Gaussian distribution, which controls the extent of blurring.

**Step 2:** Use a high-pass filter to extract the edges and texture information from the image, i.e., the high-frequency components.

(3) H=I−(G∗I)

where *I* represents the original image, G∗I represents the image after applying Gaussian filtering, and *H* represents the image containing the high-frequency components.

**Step 3:** Add the high-frequency image to the original image according to a coefficient, adjust the sharpening intensity, and merge them.

(4) B=I+αH

where α represents the sharpening intensity and *B* is the final image after sharpening.

### 2.4. Residual Network

**Figure 1:**
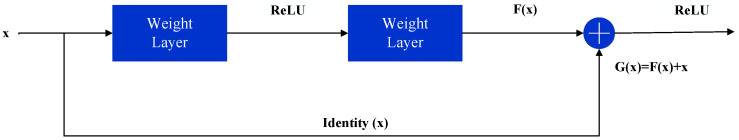
The basic architecture of ResNet.

For richer image features, a common method is to increase the network. However, as the network depth increases, the model may encounter vanishing or exploding gradient problems, which can decrease its accuracy. He et al. [24] proposed the Residual Network to simplify the training of deep networks. ResNet improves the traditional CNN and effectively addresses this issue. The structure of a residual module is illustrated in Figure 1. The output G(X) of the residual network is composed of a combination of input *x* and mapping function F(x).

### 2.5. SimAM

**Figure 2:**
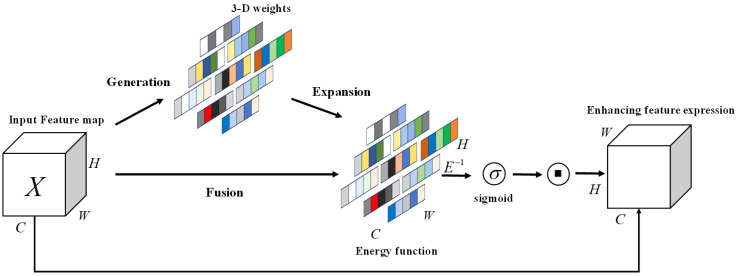
The architecture of the SimAM attention mechanism.

SimAM is an attention mechanism distinct from traditional channel attention mechanisms or spatial attention mechanisms [25]. SimAM identifies neurons with higher spatial suppression effects by defining an energy function and assigning them higher weights. Its specific framework is illustrated in Figure 2, and the energy function is expressed as follows:

(5) $$e_{t}\left(w_{t},b_{t},y,x_{i}\right)={\left(y_{t}-\hat{t}\right)}^{2}+\frac{1}{M-1}\sum_{i=1}^{M-1}{\left(y_{0}-{\hat{x}}_{i}\right)}^{2}$$

where *t* and $x_{i}$ represent the target neuron and the estimated values of other neurons on a single channel of input $X\in R^{C\times H\times W}$ (*C*, *H*, and *W* denote the number of channels, height, and width, respectively, and R is the set of real numbers). $w_{t}$ and $b_{t}$ denote the weight and bias, M=H×W represents the number of neurons in that channel, and ${\hat{t}}_{i}=w_{t}t+b_{t}$ and ${\hat{x}}_{i}=w_{t}x_{i}+b_{t}$ are the linear transformations of *t* and $x_{i}$.

Introducing the regularization coefficient λ into the weights, the energy formula is as follows:

(6) $$\begin{array}{c} e_{t}\left(w_{t},b_{t},y,x_{i}\right)=\frac{1}{M-1}\sum_{i=1}^{M-1}{\left(-1-\left(w_{t}x_{i}+b_{t}\right)\right)}^{2}+{\left(1-\left(w_{t}t+b_{t}\right)\right)}^{2}+\lambda w_{t}^{2} \end{array}$$

The solutions for $w_{t}$ and $b_{t}$ are obtained as follows:

(7) $$\left\{\begin{array}{cc} w_{t} & =-\frac{2(t-\mu_{t})}{{\left(t-\mu_{t}\right)}^{2}+2\sigma_{t}^{2}+2\lambda }\\ b_{t} & =-\frac{1}{2}\left(t+\mu_{t}\right)w_{t} \end{array}\right.$$

where $\mu_{t}=\frac{1}{M-1}\sum_{i=1}^{M-1}x_{i}$ and ${\sigma_{t}}^{2}=\frac{1}{M-1}\sum_{i=1}^{M-1}{\left(x_{i}-u_{t}\right)}^{2}$ represent the mean and variance of the channel excluding the target neuron.

The final simplified minimum energy is as follows:

(8) $$e_{t}^{*}=\frac{4({\hat{\sigma }}^{2}+\lambda )}{{\left(t-u_{t}\right)}^{2}+2{\hat{\sigma }}^{2}+2\lambda }$$

where $\hat{\sigma }$ represents the covariance value. Equation (8) reveals that the smaller the energy value, the greater the separability between the target neuron and the rest of the neurons, indicating an inverse relationship between the energy value and the separability of the target neuron from the rest. Therefore, the attention parameter is denoted by $\frac{1}{e_{t}^{*}}$.

Finally, the enhanced input with attention is obtained as follows:

(9) $$\tilde{X}=sigmoid\left(\frac{1}{E}\right)⊙X$$

## 3. The Proposed Method

**Figure 3:**
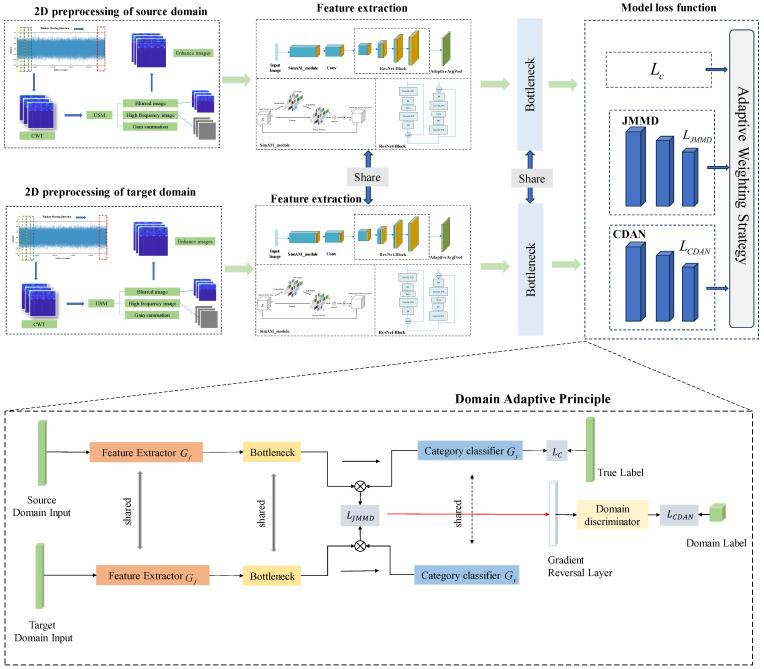
The proposed CWT-SimAM-DAMS framework.

The proposed CWT-SimAM-DAMS method is illustrated in Figure 3. The process begins with converting vibration signals into time–frequency images using the CWT algorithm. These images are then enhanced with the USM algorithm, and the enhanced data serve as input for the model. In the source domain, the feature extraction model first extracts the bearing fault features. These features are then subjected to dimensionality reduction and nonlinear transformation through a bottleneck layer, which includes a Dropout layer (p=0.5), a fully connected layer, and a ReLU activation function. The transformed features are passed through a linear classifier to compute the classification loss. Simultaneously, the features from the bottleneck layer are used with the JMMD and CDAN domain adaptation algorithms to align the joint distribution between the source and target domains and calculate the domain adaptation loss for these two algorithms. The JMMD provides a smooth and continuous alignment target, making adversarial training more stable, while the CDAN captures complex nonlinear distribution differences through adversarial learning. Additionally, the proposed weight adaptive algorithm can adjust the weights of each part in real time based on the losses from the classification, JMMD, and CDAN during model training, achieving the optimal fault monitoring state.

### 3.1. Data Processing Based on Unsharp Masking and Continuous Wavelet Transform

The core principle in information theory is that information inevitably suffers loss or degradation during transmission. Therefore, when converting one-dimensional raw data into two-dimensional images, some loss of data information is unavoidable. This paper adopts a 50% data overlap strategy [26] when generating CWT images effectively. The specific procedure is as follows. First, calculate the number of samples per cycle based on the following equation:

(10) $$N_{\min}=\frac{f_{Z}}{r/60}$$

where $f_{Z}$ represents the sampling frequency of the vibration signal [27], and *r* is the rotation speed of the bearing. Therefore, the minimum number of samples for the bearing is calculated as $N_{\min}$. However, to maintain the completeness of the sampling data, we consider $N\geq 1.5\;N_{\min}$. Then, the data are segmented through a sliding window, where the moving step size of the data segmentation window is half the number of samples per cycle. Continue this process until the end of the data is reached. This procedure is illustrated in Figure 3. After the data are segmented using a sliding window, the steps for image processing are as follows:

**Step 1:** Normalize the segmented data according to

(11) f(x)=(x−min)/(max−min)

**Step 2:** Apply the CWT algorithm to the data to transform them into a two-dimensional time–frequency image.

**Step 3:** Set different values for the key parameters and the USM algorithm and enhance each image generated by the CWT algorithm using the USM algorithm.

**Step 4:** Conduct comparative experiments on the images generated by the USM algorithm under different parameters, and select the images processed with the parameters yielding the highest fault diagnosis accuracy as the experimental input data.

### 3.2. Domain Adaptation Model Based on SimAM and ResNet

#### 3.2.1. Residual Network Integrated with SimAM

**Figure 4:**
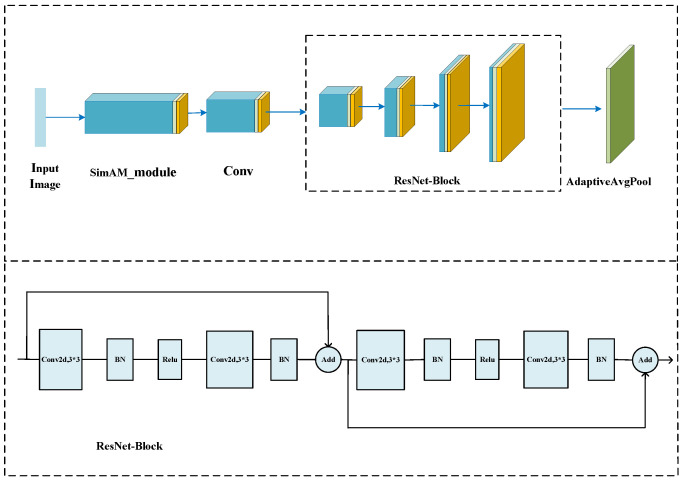
Residual network structure integrated with the SimAM attention mechanism.

In fault diagnosis, neural networks play a crucial role in feature extraction. However, traditional residual models often struggle to effectively identify fault features when dealing with complex time–frequency images, mainly due to their limited feature extraction capabilities. Hence, to address this issue, we propose the SimAM-ResNet model, which relies on ResNet as the backbone. ResNet addresses the vanishing gradient problem in deep networks by introducing residual connections, enabling easier training and optimization. Specifically, ResNet adds residual connections across the layers by directly adding the input signal to the output signal during forward propagation, thus implementing “skip connections”. This connection method enables the network to learn more accurate feature representations and significantly reduces training errors. Additionally, ResNet employs batch normalization techniques and pre-activation structures to enhance network performance and stability further. Moreover, the SimAM attention mechanism is introduced on this basis. SimAM determines attention weights by computing similarity scores between elements in the input sequence. Specifically, it calculates the similarity of each element in the input sequence to other elements, typically using the similarity or dot product operations. Then, for each element, it weights and sums the other elements based on their similarity scores to obtain the attention representation of that element. SimAM is unique because it introduces a similarity threshold which automatically filters out low-quality elements, reducing the impact of noise and redundant information. Thus, SimAM improves the robustness and generalization ability of the fault diagnosis model, which is used for refining feature mapping. The specific network structure of the fault classification module is presented in Figure 4 and Table 1.

#### 3.2.2. Joint Maximum Mean Discrepancy

The Maximum Mean Discrepancy [28] is a non-parametric metric for evaluating the difference in distributions of different datasets. It operates by mapping the feature representations of the source and target domains into the Regenerative Kernel Hilbert Space (RKHS), where the distribution discrepancy is determined by calculating the marginal distributions $P(X_{s})$ and $Q(X_{t})$ between the two domains. The MMD is defined as follows:

(12) $$MMD^{2}\left(P,Q\right)=\underset{\left||\phi \right|{|}_{H}\leq 1}{\sup}||E\left[\phi \left(x^{s}\right)\right]-E\left[\phi \left(x^{t}\right)\right]{\left|\right|}_{H}^{2}$$

where sup denotes the supremum, ϕ represents the mapping function, which maps the original dataset into the reproducing kernel Hilbert space, H denotes the reproducing kernel Hilbert space, and the subscript $\left||\phi \right|{|}_{H}\leq 1$ indicates that the norm of the function in the Hilbert space is less than or equal to 1. The empirical estimate of the MMD is given by

(13) $$\begin{array}{c} MMD^{2}\left(P,Q\right)=\left|\right|\frac{1}{n_{s}}\sum_{i=1}^{n_{s}}\phi (x_{i}^{s})-\frac{1}{n_{t}}\sum_{j=1}^{n_{t}}\phi \left(x_{j}^{t}\right){\left|\right|}_{H}^{2}\\ =\left|\right|\frac{1}{{n_{s}}^{2}}\sum_{i=1}^{n_{s}}\sum_{j=1}^{n_{s}}k(x_{i}^{s},x_{j}^{s})-\frac{2}{n_{s}n_{t}}\sum_{i=1}^{n_{s}}\sum_{j=1}^{n_{t}}k(x_{i}^{s},x_{j}^{t})+\frac{1}{{n_{t}}^{2}}\sum_{i=1}^{n_{t}}\sum_{j=1}^{n_{t}}k(x_{i}^{t},x_{j}^{t})|{|}_{H} \end{array}$$

where k(·,·) is the kernel function, and $k\left(x_{i},y_{i}\right)=\exp\left(-{\left|\left|x_{i}-y_{i}\right|\right|}^{2}/\left(2\sigma^{2}\right)\right)$.

The MMD, serving as a kernel-based two-sample test statistic, is extensively utilized to assess the distinction between marginal distributions but has not been employed to gauge the difference between joint distributions. Moreover, the MMD exhibits limited domain adaptation capability under complex multimodal conditions, and optimizing kernel parameters poses challenges. Therefore, the JMMD [29] is proposed by considering the empirical joint distributions $P(X_{s},Y_{s})$ and $Q(X_{t},Y_{t})$ between the two domains. The JMMD is defined as follows:

(14) $$L_{JMMD}\left(P,Q\right)=\left|\right|E_{P}\left({⊗}_{l=1}^{\left|L\right|}\phi^{l}\left(z_{l}^{s}\right)\right)-E_{Q}\left({⊗}_{l=1}^{\left|L\right|}\phi^{l}\left(z_{l}^{t}\right)\right){\left|\right|}_{{⊗}_{l=1}^{\left|L\right|}H^{l}}^{2}$$

where ${⊗}_{l=1}^{\left|L\right|}\phi^{l}\left(z_{l}\right)=\phi^{1}\left(z_{1}\right)⊗\cdots ⊗\phi^{\left|L\right|}\left(z_{\left|L\right|}\right)$, and $z_{l}^{s}$ represents the output of the activation function of the *l*-th layer of the network.

#### 3.2.3. Conditional Adversarial Domain Adaption

A DANN is a domain adaptive network model based on adversarial concepts. DANNs optimize learning through adversarial training between a feature extractor and a domain classifier. During the training process, domain adaptation is embedded into the model’s learning, enabling the model to extract and recognize domain-invariant features. The Category classifier trained based on adversarial concepts demonstrates good generalization in the target domain. However, DANNs do not consider the joint distribution of features and labels, which can lead to the neglect of class-specific features during training. Additionally, when the data distribution exhibits a multimodal structure, focusing solely on feature distribution makes it challenging for DANNs to accurately align the source and target domains. Long et al. [30] proposed a Conditional Domain Adversarial Network. The CDAN divides the entire network structure into three modules: a feature extractor, a Category classifier, and a domain discriminator. The CDAN addresses the problem of the DANN neglecting the joint distribution of features and labels by introducing a multilinear conditioning mechanism. Specifically, it optimizes the joint distribution of features f and labels g through multilinear mapping, thereby considering the joint distribution of features and labels. $T_{⊗}\left(f,g\right)$ and $T_{⊙}\left(f,g\right)$ are the multilinear mapping methods proposed by the CDAN. When $d_{f}\times d_{g}\leq 4096$, the CDAN takes $T_{⊗}\left(f,g\right)$ as input for the domain discriminator. When $d_{f}\times d_{g}>4096$, to avoid the dimensionality explosion, the CDAN randomly selects certain dimensions of the features and labels for multilinear mapping. In this case, the CDAN takes $T_{⊙}\left(f,g\right)$ as the input for the domain discriminator. The multilinear mapping method can capture the distribution characteristics of multimodal complex data. The loss function of the CDAN can be expressed as

(15) $$L_{CDAN}\left(\theta_{f},\theta_{d}\right)=-E_{x_{i}^{s}\in D_{s}}\;log\;\left[D\left(f_{i}^{s},g_{i}^{s}\right)\right]-E_{x_{i}^{t}\in D_{t}}\;log\;\left[1-D\left(f_{i}^{t},g_{i}^{t}\right)\right]$$

where $f_{i}^{s}=G_{f}\left(x_{i}^{s},\theta_{f}\right)$, $g_{i}^{s}=G_{c}\left(G_{f}\left(x_{i}^{s},\theta_{f}\right)\right)$, $D\left(f,g\right)=G_{d}\left(f⊗g,\theta_{d}\right)$.

Substituting $f_{i}^{s}$, $g_{i}^{s}$, and D(f,g) into Equation (15), the loss function of the CDAN can be expressed as

(16) $$\begin{array}{c} L_{CDAN}\left(\theta_{f},\theta_{d}\right)=-E_{x_{i}^{s}\in D_{s}}\;\log\left[G_{d}\left(G_{f}\left(x_{i}^{s}\right)⊗G_{c}\left(G_{f}\left(x_{i}^{s}\right)\right)\right)\right]\\ -E_{x_{i}^{t}\in D_{t}}\log\left[1-G_{d}\left(G_{f}\left(x_{i}^{t}\right)⊗G_{c}\left(G_{f}\left(x_{i}^{t}\right)\right)\right)\right] \end{array}$$

#### 3.2.4. Domain Adaptation Based on Adaptive Weighting Strategy

This paper proposes a domain adaptation method that improves the accuracy of cross-domain fault diagnosis by enabling the model to reduce marginal distribution discrepancies like the JMMD and achieve global domain alignment like the CDAN. Additionally, this paper designs an adaptive weighting strategy based on the principle that the parts of the loss function with larger values should receive more attention during the training process. The objective of this strategy is to allocate higher weights to objectives that are difficult to achieve in the current stage of the model, thereby prioritizing these parts during training. To ensure that the model learns effective source domain features in the early stages of training and improves domain adaptation ability in the later stages during target optimization, the final loss function $L_{JMMD}$ is obtained by multiplying the JMMD loss function by a parameter $\lambda_{JMMD}$. The CDAN requires minimizing the label classification loss and maximizing the domain classification loss during the optimization process. To eliminate the simultaneous maximization and minimization optimization problem, a Gradient Reversal Layer (GRL) is introduced between the feature extractor and the domain discriminator. Specifically, during forward propagation, the GRL does not perform any operation and passes the features normally through the network. During backward propagation, the GRL takes the gradient from the subsequent network, multiplies it by the parameter $-\lambda_{CDAN}$, and passes it to the previous layer. Through the above operations, the final loss function of the CDAN is obtained as $L_{CDAN}$. To effectively integrate the JMMD and CDAN, we introduce three key weights: the classifier weight $W_{c}$, distance weight $W_{JMMD}$, and adversarial weight $W_{CDAN}$. The adaptive weighting strategy dynamically adjusts these weights in real time based on the model’s performance during training and optimization objectives. Algorithm 1 describes the process of the CWT-SimAM-DAMS model training. The overall loss function of the CWT-SimAM-DAMS model can be expressed as

(17) $$\begin{array}{c} L=W_{c}L_{c}+W_{JMMD}L_{JMMD}+W_{CDAN}L_{CDAN} \end{array}$$

(18) $$W_{classifier}^{k}=\frac{3\times L_{c}^{k}}{L_{c}^{k}+L_{JMMD}^{k}+L_{CDAN}^{k}}$$

(19) $$W_{JMMD}^{k}=\frac{3\times L_{JMMD}^{k}}{L_{c}^{k}+L_{JMMD}^{k}+L_{CDAN}^{k}}$$

(20) $$W_{CDAN}^{k}=\frac{3\times L_{CDAN}^{k}}{L_{c}^{k}+L_{JMMD}^{k}+L_{CDAN}^{k}}$$

where L represents the overall loss function of the SimAM-DAMS model, and $L_{c}$ is the classification loss of the model. $W_{c}^{k}$ represents the weight corresponding to $L_{c}$ at the *K*-th epoch. $L_{JMMD}$ is the loss function of the JMMD algorithm, $W_{JMMD}^{k}$ represents the weight corresponding to $L_{JMMD}$ at the *K*-th epoch. $L_{CDAN}$ is the loss function of the CDAN algorithm. $W_{CDAN}^{k}$ represents the weight corresponding to $L_{CDAN}$ at the *K*-th epoch. In this paper, there are three loss functions. To ensure that the sum of the weights is a fixed parameter of 3, each weight is multiplied by 3, which has no special significance.
**Algorithm** **1:**  CWT-SimAM-DAMS algorithm.**Input:** epoch: max interation;*D*: CWT-USM images;Randomly initialized network parameter parameterized by θ;**Training:**set n = 0,**While** n < epoch **do**   **for** each batch in *D* **do**      obtain SimAM-RseNet results: f=F(X)      obtain classification loss      obtain JMMD loss by Equation (14) and CDAN loss by Equation (16)      calculate CWT-SimAM-DAMS loss by Equation (17)      obtain Predicted results: G(z)      update θ by using the method of gradient descent;      set n++;   **end****Output:** Predicted label y=G(z)

## 4. Data Description

The experimental platform involves a Windows 11 64-bit operating system using a 13th Gen Intel(R) Core(TM) i9-13900HX at 2.20 GHz and an NVIDIA GeForce RTX 4060 laptop GPU. The program runs in the PyCharm 2023.3.4 ×64 environment.

### 4.1. Dataset Introduction

This paper primarily utilizes two publicly available bearing fault datasets: the Case Western Reserve University bearing dataset and the dataset from the laboratory of the University of Padova. The number of epochs for the CWRU dataset is 80, and, for the PU dataset, it is 800. According to Equation (10), the signal period sampling points are 800 for the CWRU dataset and 3840 for the PU dataset. The data are split into training and testing data with a ratio of 75:25. Below is a detailed introduction to the datasets.

#### 4.1.1. Case Western Reserve University Dataset

**Figure 5:**
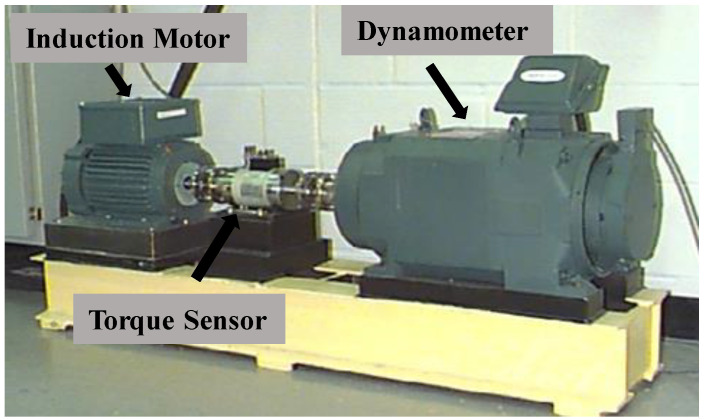
CWRU testing platform.

The CWRU collected vibration acceleration data [31] from the motor drive and fan end (Figure 5). The dataset includes normal bearing and faulty bearing operation data. This paper utilizes a sample frequency of 12 kHz for the faulty samples from the drive end.

The bearing speed is categorized into four speeds, labeled as “0, 1, 2, 3”, with different loads under each speed. The data are divided into four operating conditions, as reported in Table 2. The CWRU dataset comprises 10 bearing health conditions, including one normal and three types of faults. “IF” represents an inner ring fault, “BF” represents a ball fault, “OF” stands for outer ring fault, and “NA” represents normal bearings, as presented in Table 3. The transfer task 0-1 represents the migration from source domain operating condition 0 to target domain operating condition 1.

#### 4.1.2. Paderborn University Dataset

**Figure 6:**
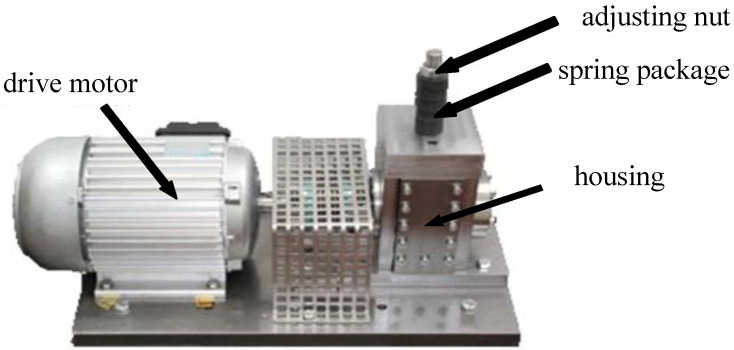
PU testing platform.

The PU dataset [32] contains two sets of data: an artificial dataset and an actual bearing damage dataset. This paper selects the actual bearing damage data collected from an accelerated life experiment. The experimental setup [33] is illustrated in Figure 6. The electric motor comprises a drive motor, adjusting nut, spring package, and housing. The vibration acceleration signal sampling frequency for the PU dataset is 64 kHz. Based on the changes in load, radia, and speed in the PU dataset, this paper selects three working conditions for the motor, as reported in Table 4. Six transfer learning tasks are constructed accordingly. This paper investigates transfer learning tasks under different operating conditions using data from 13 bearings damaged due to accelerated life experiments. Table 5 provides the classification information.

### 4.2. Experimental Parameter Settings

This paper employs the Adam algorithm as the optimizer. The $\lambda_{CDAN}$ and $\lambda_{JMMD}$ settings for this article are as follows:

(21) $$\lambda_{CDAN}=1,\lambda_{JMMD}=\frac{2}{1+e^{-10\times \left(\frac{\mathrm{current}\_\mathrm{epoch}-\mathrm{middle}\_\mathrm{epoch}}{\max\_\mathrm{epoch}-\mathrm{middle}\_\mathrm{epoch}}\right)}}-1$$

where middle_epoch is set to 0. Different datasets have varying maximum numbers of epochs, and the parameter max_epoch differs accordingly. The maximum number of epochs for the CWRU dataset is 80, and, for the PU dataset, it is 800.

When current_epoch∈[0,40), the learning rate is set to $10^{-3}$. When current_epoch∈[40,60), the learning rate is set to $10^{-4}$. When current_epoch∈[60,max_epoch), the learning rate is set to $10^{-5}$.

## 5. Experimental Verification

### 5.1. Experiment on Unsharp Mask Parameter Settings

We selected nine values for the σ and λ parameters in the Unsharp Masking algorithm based on reference [34], and the experiments were conducted on the CWRU dataset. The ResNet model was utilized, and each experiment was repeated five times. Table 6 reports the corresponding results. This demonstrates the feasibility of the USM algorithm.

By analyzing Table 6, it can be seen that, after applying the USM algorithm, the overall performance of fault diagnosis improved. In transfer task 2-3, the accuracy of all nine parameter configurations selected in this study was higher than the results using the original CWT images. Moreover, different parameter configurations had a certain impact on the final results. Specifically, when the parameters were set to σ=1.0, λ=1.5, the algorithm performed best, achieving an average accuracy of 86.59%, which is higher than the average accuracy of 84.89% achieved without using the USM algorithm. σ=1.0 and λ=1.5 represent a moderately blurred and strongly enhanced image edge and detail processing in sharpening. While reducing minor noise, it also avoids losing too many detailed features. This parameter setting makes the fault features more pronounced without excessively emphasizing noise, effectively balancing the signal-to-noise ratio and showcasing the frequency and time information of CWT images at different scales. Therefore, σ=1.0, λ=1.5 were selected as the parameter settings for the Unsharp Masking algorithm.

### 5.2. Comparative Experiment of Image Processing Method

**Figure 7:**
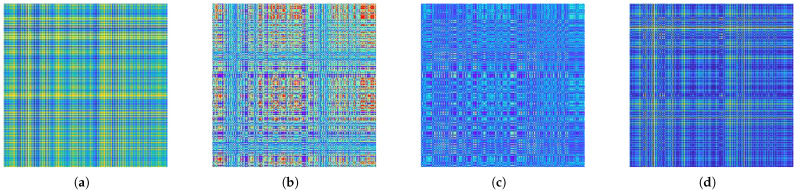
Data preprocessing 2D images: (**a**) Gramian Angular Difference Fields; (**b**) Recurrence Plot; (**c**) Markov Transition Fields; (**d**) Gramian Angular Summation Fields.

Comparative experiments were conducted on the CWRU dataset to validate the effectiveness of the proposed image extraction method (CWT-USM) by combining the CWT and USM in the signal feature extraction process. In the experiment, several different image transformation methods [35] were selected for comparison, including Gramian Angular Summation Fields (GASF), Gramian Angular Difference Fields (GADF), Recurrence Plot (RP), and Markov Transition Fields (MTF) methods. The corresponding two-dimensional images are shown in Figure 7.

**Figure 8:**
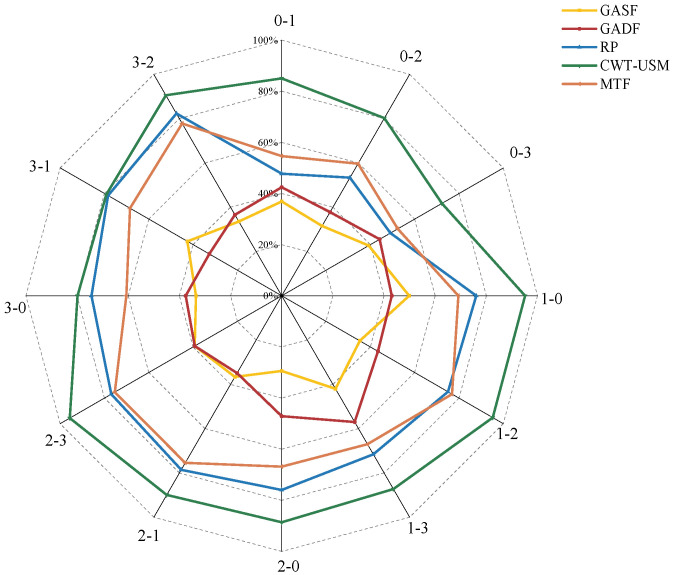
Experimental results of comparison between different two-dimensional images.

The ResNet model was selected for the experiments. Where 50% data overlap was selected for all image processing methods, the signal period sampling points were the same as that of the CWT algorithm. Figure 8 depicts the results after conducting five experiments for each method and averaging the results. By observing the experimental results, it is evident that the accuracy of images processed using GASF, GADF, RP, and MTF methods in the transfer task 0-1 was below 60%. In contrast, the accuracy of images processed with the CWT-USM method in transfer task 0-1 was 85.04%, which is a 30.4% improvement compared to the second-highest accuracy achieved by MTF (54.64%), showing a significant enhancement. Additionally, in other transfer tasks, the accuracy of the RP and MTF was significantly improved compared to that of GASF and GADF, but their accuracy was still lower than that of the CWT-USM method proposed in this study. The results indicate that the CWT-USM method can extract richer and more accurate data features, significantly improving the accuracy of bearing fault diagnosis.

### 5.3. Comparative Experiments with Different Dimensional Inputs

To compare the impact of different dimensional inputs on fault diagnosis outcomes, we evaluated the original one-dimensional (1D) time domain signal, the 1D frequency domain signal processed by FFT, and the proposed CWT-USM method, which includes time–frequency domain information. The experiments were conducted using the ResNet model, and the results are shown in Table 7.

The results indicate that CWT-USM outperformed the 1D frequency domain input across all transfer tasks. Although the accuracy of CWT-USM was slightly lower in transfer tasks 0-3, 1-2, 1-3, and 2-1 compared to the one-dimensional time domain input, the overall average accuracy of CWT-USM was higher. Specifically, the CWT-USM method improved the average accuracy by 3.61% compared to the 1D time domain input and by 14.46% compared to the 1D frequency domain input.

These experimental results demonstrate the superiority of using CWT-USM as input. By encompassing both frequency domain and time domain information relating to the vibration signal, CWT-USM provides richer feature information, leading to better fault diagnosis performance.

### 5.4. Comparative Experiments on Different Domain Adaptation Strategies

**Figure 9:**
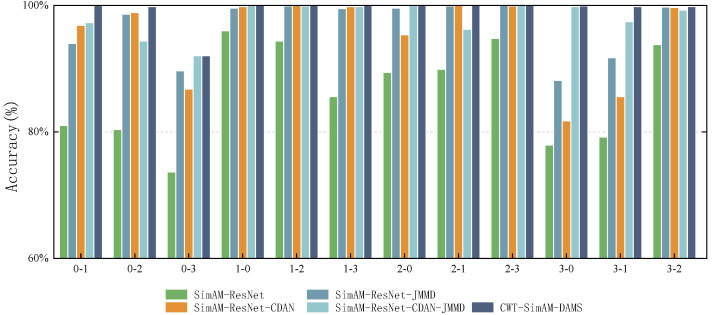
Average accuracy (%) of different domain adaptation methods on the CWRU dataset.

**Figure 10:**
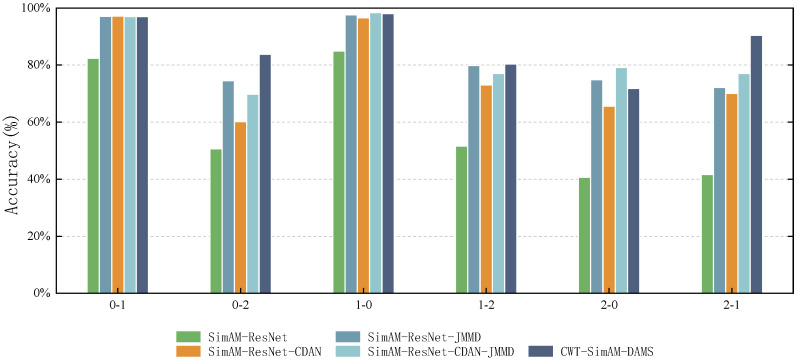
Average accuracy (%) of different domain adaptation methods on the PU dataset.

To enhance the persuasiveness and general applicability of the experiments, this study introduced the PU bearing and the existing CWRU datasets. The experiments extensively compared several transfer strategies, including the baseline model without any transfer strategy (SimAM-ResNet), utilizing the Conditional Domain Adaptation Network (SimAM-ResNet-CDAN), utilizing the Maximum Mean Discrepancy (SimAM-ResNet-JMMD), a model combining CDAN and JMMD but without the adaptive weighting algorithm (SimAM-ResNet-CDAN-JMMD), and the proposed method (CWT-SimAM-DAMS). The experiments were repeated five times, and the results on the CWRU and PU datasets are presented in Table 8 and Table 9, as well as Figure 9 and Figure 10, respectively.

On the CWRU dataset, compared to SimAM-ResNet without domain adaptation algorithm or SimAM-ResNet-CDAN and SimAM-ResNet-JMMD using one domain adaptation algorithm alone, SimAM-ResNet-CDAN-JMMD, a method that combines two domain adaptation algorithms, had an improved average fault diagnostic accuracy. However, there was still be a problem where the accuracy rate decreased in migration tasks compared to when using a domain adaptation algorithm alone, e.g., migration task 0-2. The proposed method, CWT-SimAM-DAMS, achieves an accuracy rate that is greater than or equal to that of other domain adaptation algorithms across all migration tasks. Additionally, this method addresses the decreased accuracy of the SimAM-ResNet-CDAN-JMMD method compared to the SimAM-ResNet-CDAN and SimAM-ResNet-JMMD methods on migration task 0-2. On the PU dataset, the proposed method showed a significant improvement in migration task 0-2 and migration task 2-1. Although it decreased in migration tasks 0-1, 1-0, and 2-0, the conditions 0-2 and 2-1 were improved by 14.03% and 13.42%, respectively, compared to the SimAM-ResNet-CDAN-JMMD method. Thus, it significantly improves the average fault diagnosis accuracy. Compared to other domain adaptation algorithms, the proposed CWT-SimAM-DAMS method exhibits stronger adaptability and accuracy. Because it adjusts the optimization objectives in real time, by comprehensively considering three optimization objectives, the classification, JMMD, and CDAN, this method reduces the distribution differences between the source and target domains, enhancing the model’s ability to diagnose bearing failures.

### 5.5. Model Comparison Experiment

**Figure 11:**
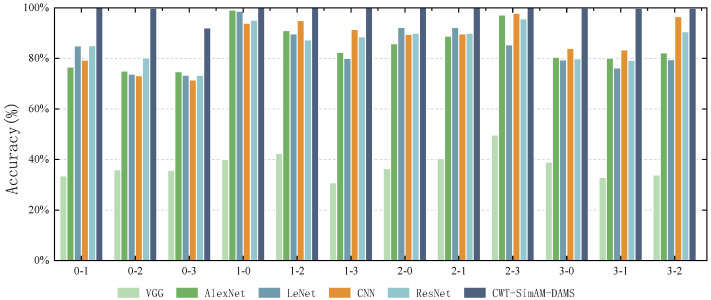
Average diagnostic accuracy (%) of different models on the CWRU dataset.

**Figure 12:**
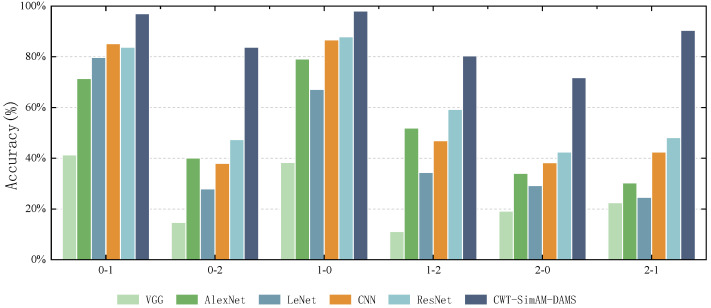
Average diagnostic accuracy (%) of different models on the PU dataset.

**Figure 13:**
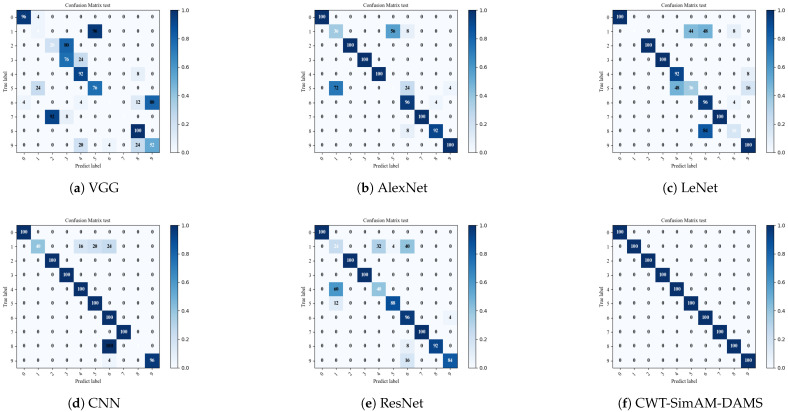
Visualization of confusion matrix for different models in the target domain of CWRU dataset 0-1 migration task.

**Figure 14:**
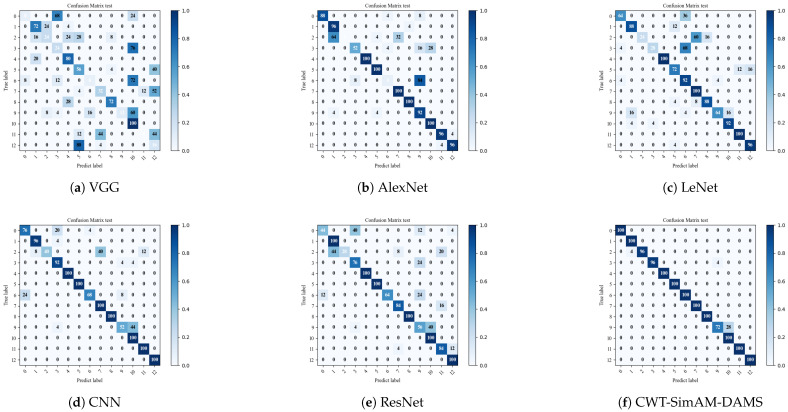
Visualization of confusion matrix for different models in the target domain of PU dataset 0-1 migration task.

To verify the feasibility of our proposed bearing fault diagnosis model compared to other bearing fault diagnosis models, we selected several common algorithms and models in bearing fault diagnosis for comparative verification. Each model was applied five times to obtain the average diagnostic accuracy. Figure 11 and Figure 12, as well as Table 10 and Table 11, present the experimental results comparing the CWT-SimAM-DAMS model with the competitor models. Table 12 and Table 13 present the training and testing times of different models. Additionally, the performance of each method was assessed through confusion matrices, as shown in Figure 13 and Figure 14.

The experimental results show that the CWT-SimAM-DAMS model achieved an average accuracy of 99.29% on the CWRU dataset and 86.93% on the PU dataset. Compared to several traditional bearing fault diagnosis methods, the CWT-SimAM-DAMS method significantly improves average accuracy. Specifically, the accuracy of the CWT-SimAM-DAMS method on the CWRU and PU datasets was 13.56% and 25.42% higher, respectively, than that of the traditional ResNet model. Similarly, compared to the CNN model, the CWT-SimAM-DAMS method achieved an average accuracy improvement of 12.18% and 30.66% on the CWRU and PU datasets, respectively. This indicates that the CWT-SimAM-DAMS model has superior feature extraction and domain alignment capabilities.

Table 12 and Table 13 show that the ResNet model had the longest training and testing times on both the CWRU and PU datasets. Although the CWT-SimAM-DAMS model has relatively long training times compared to other models, its testing time does not significantly increase. For the CWRU dataset, the training time difference between all models was less than one minute, and the testing time of the CWT-SimAM-DAMS model was only 0.068898 min longer than the fastest AlexNet model. For the PU dataset, the training time difference between all models was within 10 min, and the testing time of the CWT-SimAM-DAMS model was only 0.4784722 min longer than the fastest CNN model. Considering that, in practical industrial applications, model training is usually conducted offline, training time is not a critical issue compared to model accuracy. Additionally, the difference in testing time between models is not significant. Taking both model accuracy and testing time into account, the CWT-SimAM-DAMS model still has a significant advantage.

### 5.6. Ablation Study

Various ablation experiments were conducted on the CWRU dataset to verify the proposed CWT-SimAM-DAMS method, referred to as Method 1 in Table 14, and the efficacy of each component. These ablation experiments involved systematically removing key modules of Method 1 and observing their impact on the final performance, thereby revealing the contributions and importance of each module.

By comparing different combinations, it was found that removing the adaptive weighting module led to a significant decrease in performance, indicating the critical importance of the adaptive weighting module for the effectiveness of the CWT-SimAM-DAMS method. Conversely, when CWT-USM was replaced with regular CWT or the Residual Network integrated with SimAM was substituted by a standard residual network, although there was a decrease in performance, the impact was relatively minor. This indicates that, while the module image processing and the residual network integrated with SimAM contribute to performance enhancement, their effect is not as pronounced as that of the weight adaptive strategy module. The complete Method 1 model outperformed all other combinations, validating that integrating all modules achieves the best performance.

## 6. Conclusions

This study proposes a bearing fault diagnosis method based on SimAM and an adaptive weighting transfer strategy. The proposed method transforms one-dimensional vibration time series signals of bearing faults into CWT images and enhances the detailed features of the images using the USM algorithm, facilitating feature extraction by the model. Integrating the SimAM attention mechanism into the residual network enhances the model’s feature extraction capability in the source domain. Additionally, by combining the JMMD and CDAN algorithms and employing a weight adaptive strategy, the domain adaptation transfer capability of the model is strengthened.

The proposed method is validated on both the CWRU and PU datasets, achieving an accuracy of 99.29% on the CWRU dataset and 86.93% on the PU dataset, representing a significant improvement compared to other models. Moreover, ablation experiments conducted on the CWRU dataset verify the importance and effectiveness of each component. The experimental results demonstrate that this method effectively reduces the distribution difference between the source and target domains, improving fault diagnosis accuracy.

In future research, further optimization of the model architecture will be pursued to enhance its generalization, and application in more realistic industrial scenarios will be explored. Additionally, refinement of model parameters will be conducted to improve both training and testing times for fault diagnosis while maintaining the model’s accuracy.

## Figures and Tables

**Table 1:** Parameters of the residual network structure integrated with the SimAM attention mechanism.

Layer		Parameter	Output Size
SimAM_module	\	\	32 × 32 × 3
Conv2d	Conv2d	kernel_size = 7, stride = 2, pad = 3	16 × 16 × 64
Layer1	Conv2d	kernel_size = 3, stride = 1, pad = 1	16 × 16 × 64
	Conv2d	kernel_size = 3, stride = 1, pad = 1	16 × 16 × 64
	Conv2d	kernel_size = 3, stride = 1, pad = 1	16 × 16 × 64
	Conv2d	kernel_size = 3, stride = 1, pad = 1	16 × 16 × 64
Layer2	Conv2d	kernel_size = 3, stride = 2, pad = 1	8 × 8 × 128
	Conv2d	kernel_size = 3, stride = 1, pad = 1	8 × 8 × 128
	Conv2d	kernel_size = 3, stride = 1, pad = 1	8 × 8 × 128
	Conv2d	kernel_size = 3, stride = 1, pad = 1	8 × 8 × 128
Layer3	Conv2d	kernel_size = 3, stride = 2, pad = 1	4 × 4 × 256
	Conv2d	kernel_size = 3, stride = 1, pad = 1	4 × 4 × 256
	Conv2d	kernel_size = 3, stride = 1, pad = 1	4 × 4 × 256
	Conv2d	kernel_size = 3, stride = 1, pad = 1	4 × 4 × 256
Layer4	Conv2d	kernel_size = 3, stride = 2, pad = 1	2 × 2 × 512
	Conv2d	kernel_size = 3, stride = 1, pad = 1	2 × 2 × 512
	Conv2d	kernel_size = 3, stride = 1, pad = 1	2 × 2 × 512
	Conv2d	kernel_size = 3, stride = 1, pad = 1	2 × 2 × 512
AdaptiveAvgPool	\	\	1 × 1 × 512

**Table 2:** CWRU dataset operating conditions and data splitting.

Task		0	1	2	3
Speed (rpm)		1797	1772	1750	1730
Load (HP)		0HP	1HP	2HP	3HP
Dataset	train	75	75	75	75
	test	25	25	25	25

**Table 3:** CWRU dataset fault condition information.

Class Label	0	1	2	3	4	5	6	7	8	9
Fault Location	NA	IF	BF	OF	IF	BF	OF	IF	BF	OF
Fault Size (mils)	0	7	7	7	14	14	14	21	21	21

**Table 4:** PU dataset operating conditions and data splitting.

Task		0	1	2
Load (Nm)		0.7	0.1	0.7
Radia (N)		1000	1000	400
Speed (rpm)		1500	1500	1500
Dataset	train	75	75	75
	test	25	25	25

**Table 5:** PU dataset fault condition information.

Class Lable	0	1	2	3	4	5	6	7	8	9	10	11	12
Bearing Code	KA04	KA15	KA16	KA22	KA30	KB23	KB24	KB27	KI14	KI16	KI17	KI18	KI21
Bearing Element	OR	OR	OR	OR	OR	IR(+OR)	IR(+OR)	IR	IR	IR	IR	IR	IR
Combination	S	S	R	S	R	M	M	M	M	S	R	S	S

S: single damage; M: multiple damage; R: repetitive damage; IR: inner ring; OR: outer ring.

**Table 6:** Experimental results of USM algorithm parameter comparison.

Parameters of USM	0-1	0-2	0-3	1-0	1-2	1-3	2-0	2-1	2-3	3-0	3-1	3-2	Average
Not using USM	79.96	76.4	68.88	92.72	94.8	84.84	88.12	90.48	93.7	80.56	79.12	89.12	**84.89**
σ=0.7,α=1.0	69.04	73.6	71.76	92.48	95.52	90.24	88.32	89.84	95.68	83.12	78.96	91.6	85.01
σ=1.0,α=0.5	85.2	76.72	72.16	90.64	95.76	90.92	86.16	91.84	97.12	80.48	78	89.28	86.19
σ=1.0,α=0.8	76.88	81.16	71.04	87.44	91.28	86.8	87.6	88.4	96.16	81.04	77.88	89.12	84.57
σ=1.0,α=1.0	80.96	76.16	70.32	90.6	95.28	88.48	87.84	90.08	97.84	77.76	79.12	88.4	85.24
σ=1.0,α=1.3	80	75.28	72.76	94.24	93.68	90	88.56	89.76	97.44	79.84	79.2	89.84	85.88
σ=1.0,α=1.5	85.04	80.24	72.32	95.12	95.28	87.28	88.6	89.92	95.64	79.84	79.2	90.56	**86.59**
σ=1.3,α=1.0	74.56	78.56	73.44	89.28	94.48	83.68	88.8	89.84	98.68	78.56	80.16	91.84	85.16
σ=1.3,α=1.5	79.56	76.186	70.32	91.12	91.574	85.866	89.76	91.04	95.84	79.84	78.64	88.24	84.83
σ=1.5,α=1.0	78.56	72.32	68.48	91.44	97.76	87.28	91.52	88.88	95.48	80.4	80.08	90.48	85.22

**Table 7:** Comparative experiments with different dimensional inputs for the CWRU dataset.

Methods	0-1	0-2	0-3	1-0	1-2	1-3	2-0	2-1	2-3	3-0	3-1	3-2	Average
1D time domain input	80.32	79.84	**75.65**	81.77	**99.84**	**96.69**	74.19	**93.87**	94.68	69.11	72.34	77.42	82.98
1D frequency domain input	73.22	67.31	54.48	72.45	79.01	81.34	67	83.43	82.33	55.78	70.35	78.86	72.13
CWT-USM	**85.04**	**80.24**	72.32	**95.12**	95.28	87.28	**88.6**	89.92	**95.64**	**79.84**	**79.2**	**90.56**	**86.59**

**Table 8:** Experimental results of comparing different domain adaptation strategies on the CWRU dataset.

Methods	0-1	0-2	0-3	1-0	1-2	1-3	2-0	2-1	2-3	3-0	3-1	3-2	Average
SimAM-ResNet	81.04	80.4	73.68	96	94.4	85.6	89.44	89.92	94.8	77.92	79.2	93.8	86.28
SimAM-ResNet-JMMD	94	98.64	89.68	99.6	99.92	99.52	99.6	99.92	**100**	88.16	91.76	99.76	96.71
SimAM-ResNet-CDAN	96.88	98.88	86.8	99.84	**100**	99.84	95.36	**100**	99.92	81.76	85.6	99.68	95.38
SimAM-ResNet-CDAN-JMMD	97.28	94.4	**92.08**	**100**	99.92	99.84	**100**	96.24	**100**	95.36	97.44	99.28	97.65
CWT-SimAM-DAMS	**100**	**99.84**	**92.08**	**100**	**100**	**100**	**100**	**100**	**100**	**99.92**	**99.84**	**99.84**	**99.29**

**Table 9:** Experimental results of comparing different domain adaptation strategies on the PU dataset.

Methods	0-1	0-2	1-0	1-2	2-0	2-1	Average
SimAM-ResNet	82.4	50.65	84.92	51.63	40.74	41.66	58.67
SimAM-ResNet-JMMD	97.11	74.52	97.66	79.82	74.89	72.18	82.7
SimAM-ResNet-CDAN	**97.17**	60.18	96.55	73.05	65.6	70.09	77.11
SimAM-ResNet-CDAN-JMMD	97.05	69.78	**98.4**	77.05	**79.2**	77.04	83.09
CWT-SimAM-DAMS	97.05	**83.81**	98.09	**80.37**	71.81	**90.46**	**86.93**

**Table 10:** Average diagnostic accuracy (%) of different models on the CWRU dataset.

Methods	0-1	0-2	0-3	1-0	1-2	1-3	2-0	2-1	2-3	3-0	3-1	3-2	Average
VGG	33.52	36	35.76	40	42.4	30.8	36.48	40.4	49.72	38.96	32.88	33.84	37.56
AlexNet	76.54	75.04	74.72	99.12	90.96	82.4	85.84	88.8	97.12	80.48	80.16	82.24	84.45
LeNet	84.96	73.76	73.38	98.64	89.68	80	92.24	92.26	85.4	79.44	76.24	79.52	83.79
CNN	79.36	73.2	71.52	93.92	94.96	91.44	89.52	89.68	97.84	84	83.36	96.56	87.11
ResNet	85.04	80.24	72.32	95.12	95.28	87.28	88.6	89.92	95.64	79.84	79.2	90.56	86.59
CWT-SimAM-DAMS	**100**	**99.84**	**92.08**	**100**	**100**	**100**	**100**	**100**	**100**	**99.92**	**99.84**	**99.84**	**99.29**

**Table 11:** Average diagnostic accuracy (%) of different models on the PU dataset.

Methods	0-1	0-2	1-0	1-2	2-0	2-1	Average
VGG	41.34	14.74	38.36	11.08	19.16	22.548	24.54
AlexNet	71.48	40.12	79.22	51.97	34.02	30.32	51.19
LeNet	79.79	27.99	67.14	34.41	29.23	24.62	43.86
CNN	85.16	38.03	86.7	46.95	38.26	42.5	56.27
ResNet	83.82	47.38	87.95	59.26	42.48	48.16	61.51
CWT-SimAM-DAMS	**97.05**	**83.81**	**98.09**	**80.37**	**71.81**	**90.46**	**86.93**

**Table 12:** The average training and testing time of each model in the CWRU dataset.

Methods	VGG	AlexNet	LeNet	CNN	ResNet	CWT-SimAM-DAMS
Training time (min)	1.856917	1.658083	1.841167	2.258319	2.541681	2.431865
Test time (min)	0.282778	0.268042	0.316833	0.381056	0.385319	0.33694

**Table 13:** The average training and testing time of each model in the PU dataset.

Methods	VGG	AlexNet	LeNet	CNN	ResNet	CWT-SimAM-DAMS
Training time (min)	27.183528	25.044139	23.018417	23.274472	32.809889	30.7255
Test time (min)	4.0978333	3.8351389	3.8351944	3.7904167	4.8703611	4.2688889

**Table 14:** CWUR dataset ablation experiment result.

	Image Processing	Model Selection	Domain Adaptation	Accuracy												
	**CWT-** **USM**	**SimAM-** **ResNet**	**Adaptive** **Weight** **Strategy**	**0-1**	**0-2**	**0-3**	**1-0**	**1-2**	**1-3**	**2-0**	**2-1**	**2-3**	**3-0**	**3-1**	**3-2**	**Average**
Method 1	✓	✓	✓	100	99.84	92.08	100	100	100	100	100	100	99.92	99.84	99.84	99.29
Method 2	✓	✓	×	81.04	80.4	73.68	96	94.4	85.6	89.44	89.92	94.8	77.92	79.2	93.8	86.28
Method 3	✓	×	×	85.04	80.24	72.32	95.12	95.28	87.28	88.6	89.92	95.64	79.84	79.2	90.56	86.59
Method 4	✓	×	✓	96.16	90.56	93.36	99.92	99.76	99.92	97.76	98.8	100	99.92	99.9	99.8	97.92
Method 5	×	✓	×	80.08	73.5	75	93.92	94.32	84.72	85.2	89.68	94.4	78.32	77.12	85.6	84.76
Method 6	×	✓	✓	100	99.92	93.44	100	100	100	100	100	100	92.16	84.48	99.76	97.48
Method 7	×	×	×	79.96	76.4	68.88	92.72	94.8	84.84	88.12	90.48	93.7	80.56	79.12	89.12	84.89
Method 8	×	×	✓	99.92	97.52	90.08	99.84	99.84	99.76	100	100	100	95.92	85.68	99.84	97.37

✓ represents selecting the corresponding module, × represents not selecting the corresponding module.

## Data Availability

The detailed data information of this article is included in the article. For detailed information, please contact the corresponding author.
